# Supplementary material for: Evidence of Histoplasma capsulatum seropositivity and exploration of risk factors for exposure in Busia county, western Kenya: Analysis of the PAZ dataset
Source: PLoS Negl Trop Dis. 2023 May 12;17(5):e0011295. doi: 10.1371/journal.pntd.0011295 (PMC10180684; doi:10.1371/journal.pntd.0011295)
Supplement: S1 Table — Analysis of observations of bats and wild birds only. (DOCX) [file pntd.0011295.s001.docx]

**Table S1. Variables selected for data analysis, from original survey human and household survey reports [1]**.

| Category | Selected variables |
| --- | --- |
| *Histoplasma* antibody detection | - IMMY LA-*Histoplasma* test result |
| Demographic characteristics | - Respondent age - Respondent sex - What is your major occupation? |
| Disease status and clinical presentation | - HIV status - Do you smoke cigarettes? |
| Animal contact or observation | - Do you have contact with dogs? - Do you have contact with cats? - [In the last 12 months] What wildlife have you seen around the home?^1^ - In the last 12 months, have you seen rats around the home? - In the last 12 months, have you been involved with skinning dead animals within or outside the home? - In the last 12 months, have you been involved with burying dead animals within or outside the home? - In the last 12 months, have you been involved with manure preparation within or outside the home? - Involvement with manure preparation within or outside home in previous 12 months |
| Household data | - Homestead ID - Where did you obtain your water from in the last wet season? - Where did you obtain your water from in the last dry season? - How many houses (dwellings occupied by humans) are there on the compound? - Roof – how many with iron sheets/ thatch/ tiles/ other materials? - Walls: how many with mud (no bricks)/ unburnt bricks/ burnt mud bricks/ burnt bricks and cement/ mud with cement/ timber/ cement only/ stone/ other materials? - Floor: how many have earth/ cemented/ tiled/ wooden/ other materials? - What is the main cooking fuel in the household? |

^1^Analysis of observations of bats and wild birds only.

**References**

1. Fèvre EM, de Glanville WA, Thomas LF, Cook EAJ, Kariuki S, Wamae CN. An integrated study of human and animal infectious disease in the Lake Victoria crescent small-holder crop-livestock production system, Kenya. BMC Infect Dis. 2017;17: 1–14. doi:10.1186/s12879-017-2559-6
