# Supplementary material for: Evidence of Histoplasma capsulatum seropositivity and exploration of risk factors for exposure in Busia county, western Kenya: Analysis of the PAZ dataset
Source: PLoS Negl Trop Dis. 2023 May 12;17(5):e0011295. doi: 10.1371/journal.pntd.0011295 (PMC10180684; doi:10.1371/journal.pntd.0011295)
Supplement: S2 Table — NR = not recorded; ND = not determined; NA = not applicable. (DOCX) [file pntd.0011295.s002.docx]

**S2 Table. Original survey human and homestead report text, and re-coding of selected variables.**

| Variable of interest | Original survey questions | Variable type | Original survey responses | Re-coding for current analysis |
| --- | --- | --- | --- | --- |
| Age | Respondent age | Ordinal | NR; <1; 1-4; 5-9; 10-14; 15-19…80-84; 85+ | 5-14; 15-24; 25-34; 35-44; ≥45 |
| Gender | Respondent sex | Categorical | Male; Female; ND | No change (ND not recorded) |
| Occupation | What is your major occupation? | Categorical | Farmer; Trader; Shop keeper; Full time parent; Student; Driver; Other; NR | Re-categorised (**S3 Table**) |
|  | If other, please enter details of occupation | Freeform text | See **S3 Table** for re-coding of Other |  |
| Smoking behaviour | Do you smoke cigarettes? | Categorical | Daily; Weekly; Monthly; On special occasions; Previously but not anymore; Never; NR | No (Never OR Previously but not anymore OR On special occasions OR NR); Yes (Daily OR Weekly OR Monthly) |
| HIV status |  | Categorical | 0 (Negative); 1 (Positive) | No change |
| Cat and dog contact | Do you have contact with cats?  Do you have contact with dogs? | Categorical | Never; Daily; At least once a week; At least once a month; At least once a year; Used to but no longer; ND | No (Never OR Used to but no longer); Yes (Daily OR At least once a week OR At least once a month OR At least once a year); ND not recorded |
| Livestock in building | Do livestock have access to the building you sleep in? Tick all that apply. | Categorical | No; Cattle; Pigs; Goats; Sheep; Chickens; Ducks; Other; NR | No change (NR not recorded) |
| Wildlife observation | What wildlife have you seen around the home? (any non-domestic species should be listed; tick all that apply): Bats; Wild birds | Categorical | No; Yes; NR | No (No or NR); Yes |
|  | In the last 12 months, have you seen rats around the home? | Categorical | Daily; Sometimes; Never; NR | No (Never); Yes (Daily OR Sometimes); NR not recorded |
| Animal contact activities | In the last 12 months, have you been involved with skinning dead animals within or outside the home? | Categorical | No  Daily  At least once per week  At least once a month  At least once a year  Used to but not anymore  NR | No (No OR Used to but not anymore OR NR); Yes (Daily OR At least once per week OR At least once a month OR At least once a year) |
|  | In the last 12 months, have you been involved with burying dead animals within or outside the home? | Categorical | No  Daily  At least once per week  At least once a month  At least once a year  Used to but not anymore  NR | No (No OR Used to but not anymore OR NR); Yes (Daily OR At least once per week OR At least once a month OR At least once a year) |
|  | In the last 12 months, have you been involved with manure preparation within or outside the home? | Categorical | No  Daily  At least once per week  At least once a month  At least once a year  Used to but not anymore  NR | No (No OR Used to but not anymore OR NR); Yes (Daily OR At least once per week OR At least once a month OR At least once a year) |
| Water source | Where did you obtain your water from in the last wet season?  Where did you obtain your water from in the last dry season? | Categorical (per independent water source variable) | Borehole; River; Pump; Tap; Well; Spring; Other; NR | No (0); Yes (1) |
| Dwelling roof material | Roof - how many with iron sheets/ thatch/ tiles/ other materials? | Continuous | NA | No (0); Yes (≥1) |
| Dwelling wall material | Walls – how many with mud (no bricks)/ unburnt bricks/ burnt mud bricks/ burnt bricks and cement/ mud with cement/ timber/ cement only/ stone/ other materials? | Continuous | NA | No (0); Yes (≥1) |
| Dwelling floor material | Floor: how many have earth/ cemented/ tiled/ wooden/ other materials? | Continuous | NA | No (0); Yes (≥1) |
| Cooking fuel | What is the main cooking fuel in the household? | Categorical | Open fire – firewood; Open fire – charcoal; Gas stove; Jico stove; Paraffin stove; Solar stove; Electric stove; NR | Firewood open fire; Charcoal open fire; Firewood and charcoal open fire |

NR=not recorded; ND=not determined; NA=not applicable.
