# Supplementary material for: Evidence of Histoplasma capsulatum seropositivity and exploration of risk factors for exposure in Busia county, western Kenya: Analysis of the PAZ dataset
Source: PLoS Negl Trop Dis. 2023 May 12;17(5):e0011295. doi: 10.1371/journal.pntd.0011295 (PMC10180684; doi:10.1371/journal.pntd.0011295)
Supplement: S3 Table — (DOCX) [file pntd.0011295.s003.docx]

S3 Table. **Re-categorised occupations for statistical analysis.**

| Re-categorised occupation categories | Original occupation categories |
| --- | --- |
| Animal contact roles | Herds boy  Herdsman  Farmer  Animal health assistant  Housekeeping  House help (to homestead)  Full time parent |
| Building role | Mason/ masonry  Wiring  Carpenter/ carpentry  Welding  Brick maker/ brick making  House builder |
| Teacher or student | Student  Teacher |
| Trader | Trader  Shop keeper |
| Other | Catechist  Saloon  Tailor/ tailoring/ Dressmaker/ dress making  Prison Warden  Hairdresser  Accountant  Security Officer/ Security (Guard)/ Watchman  Bicycle repair  Chef  Nurse  Casual (labourer)  Chief  Juakali  Mechanic  Weaving  Driver |
| Not specified or not applicable | NA  None  Other |
