# Supplementary material for: Evidence of Histoplasma capsulatum seropositivity and exploration of risk factors for exposure in Busia county, western Kenya: Analysis of the PAZ dataset
Source: PLoS Negl Trop Dis. 2023 May 12;17(5):e0011295. doi: 10.1371/journal.pntd.0011295 (PMC10180684; doi:10.1371/journal.pntd.0011295)
Supplement: S4 Table — * p<0.05. (DOCX) [file pntd.0011295.s004.docx]

**S4 Table.** **Baseline characteristics of original sample (*n*=942) and selected sample (*n*=670) of survey respondents, and comparison of variable distribution differences using the Mann-Whitney U test.**

| Variable | Original sample, *n*=942 | Study sub-set, *n*=670 | Mann-Whitney U test *p-*value |
| --- | --- | --- | --- |
| Demographic | | | |
| Sex |  |  |  |
| Male | 450 (47.8) | 320 (47.8) | 1.00 |
| Female | 492 (52.2) | 350 (52.2) |  |
| Age category, years |  |  |  |
| 5-14 | 388 (41.2) | 272 (40.6) | 0.94 |
| 15-24 | 178 (18.9) | 133 (19.9) |  |
| 25-34 | 97 (10.3) | 72 (10.7) |  |
| 35-44 | 105 (11.3) | 76 (11.3) |  |
| ≥45 | 174 (18.5) | 117 (17.5) |  |
| Occupation |  |  |  |
| Not applicable or None | 25 (2.7) | 16 (2.4) | 0.84 |
| Animal contact roles | 337 (35.8) | 244 (36.4) |  |
| Building roles | 22 (2.3) | 16 (2.4) |  |
| Teacher or Student | 476 (50.5) | 341 (50.9) |  |
| Trader | 43 (4.6) | 28 (4.2) |  |
| Other (S3 Table) | 39 (4.1) | 25 (3.7) |  |
| Clinical | | | |
| HIV status |  |  |  |
| Negative | 874 (92.8) | 622 (92.8) | 0.24 |
| Positive | 53 (5.6) | 48 (7.2) |  |
| No answer | 15 (1.6) | - |  |
| Smoking behaviour |  |  |  |
| No | 915 (97.1) | 653 (97.5) | 0.69 |
| Yes | 27 (2.9) | 17 (2.5) |  |
| Domestic animal or livestock contact | | | |
| Dog contact |  |  |  |
| No | 147 (15.6) | 88 (13.1) | 0.17 |
| Yes | 795 (84.4) | 582 (86.9) |  |
| Cat contact |  |  |  |
| No | 162 (17.2) | 107 (16.0) | 0.52 |
| Yes | 780 (82.8) | 563 (84.0) |  |
| Poultry building access |  |  |  |
| No | 114 (12.1) | 73 (10.9) | 0.46 |
| Yes | 828 (87.9) | 597 (89.1) |  |
| Pigs building access |  |  |  |
| No | 907 (96.3) | 642 (95.8) | 0.64 |
| Yes | 35 (3.7) | 28 (4.2) |  |
| Shoats building access |  |  |  |
| No | 898 (95.3) | 639 (95.4) | 0.97 |
| Yes | 44 (4.7) | 31 (4.6) |  |
| Cattle building access |  |  |  |
| No | 900 (95.5) | 637 (95.1) | 0.66 |
| Yes | 42 (4.5) | 33 (4.9) |  |
| Burying dead animals |  |  |  |
| No | 928 (98.5) | 658 (98.2) | 0.63 |
| Yes | 14 (1.5) | 12 (1.8) |  |
| Skinning dead animals |  |  |  |
| No | 925 (98.2) | 656 (97.9) | 0.68 |
| Yes | 17 (1.8) | 14 (2.1) |  |
| Manure preparation |  |  |  |
| No | 366 (38.9) | 234 (34.9) | 0.12 |
| Yes | 576 (61.1) | 436 (65.1) |  |
| Wildlife observation around home | | | |
| Bats |  |  |  |
| No | 554 (58.8) | 321 (47.9) | <0.001* |
| Yes | 368 (39.1) | 348 (51.9) |  |
| No answer | 20 (2.1) | 1 (0.1) |  |
| Rats |  |  |  |
| No | 88 (9.3) | 61 (9.1) | 0.87 |
| Yes | 854 (90.7) | 609 (90.9) |  |
| Wild birds |  |  |  |
| No | 768 (81.5) | 554 (82.7) | 0.80 |
| Yes | 154 (16.3) | 115 (17.2) |  |
| No answer | 20 (2.1) | 1 (0.1) |  |

* *p*<0.05
