# Supplementary figures and images for: Evidence of Histoplasma capsulatum seropositivity and exploration of risk factors for exposure in Busia county, western Kenya: Analysis of the PAZ dataset
Source: PLoS Negl Trop Dis. 2023 May 12;17(5):e0011295. doi: 10.1371/journal.pntd.0011295 (PMC10180684; doi:10.1371/journal.pntd.0011295)

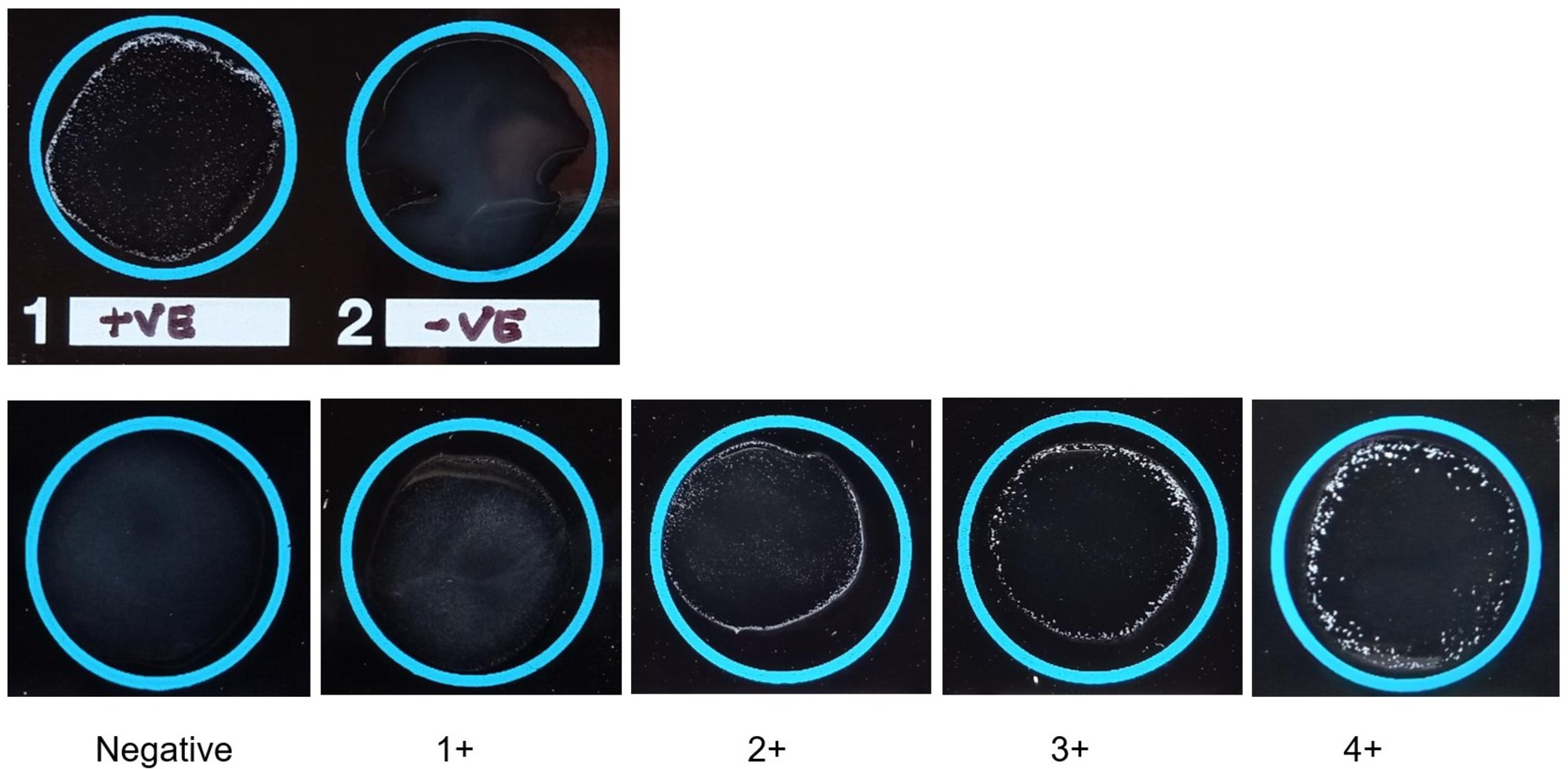

Supplement: S1 Fig — (TIFF) [file pntd.0011295.s005.tiff]

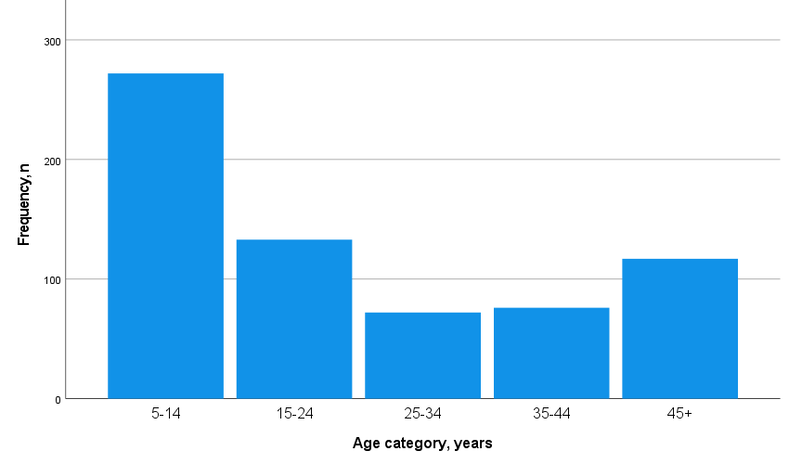

Supplement: S2 Fig — (TIFF) [file pntd.0011295.s006.tiff]
