# Supplementary material for: Evidence of Histoplasma capsulatum seropositivity and exploration of risk factors for exposure in Busia county, western Kenya: Analysis of the PAZ dataset
Source: PLoS Negl Trop Dis. 2023 May 12;17(5):e0011295. doi: 10.1371/journal.pntd.0011295 (PMC10180684; doi:10.1371/journal.pntd.0011295)
Supplement: Attachment — Submitted filename: PNTD-D-23-00017-annotated.pdf [file pntd.0011295.s007.pdf]

PLOS Neglected Tropical Diseases

Evidence of Histoplasma capsulatum seropositivity and exploration of risk factors for exposure in Busia county, western Kenya: Analysis of the PAZ dataset

--Manuscript Draft--

**Manuscript Number:**	PNTD-D-23-00017
**Full Title:**	Evidence of Histoplasma capsulatum seropositivity and exploration of risk factors for exposure in Busia county, western Kenya: Analysis of the PAZ dataset
**Short Title:**	Histoplasma capsulatum seroprevalence and risk factors in western Kenya
**Article Type:**	Research Article
**Keywords:**	Histoplasma capsulatum; seroprevalence; Kenya; human-animal-environment interface
**Abstract:**	Background<br>Despite recognition of histoplasmosis as a disease of national public health concern in Kenya, the burden of Histoplasma capsulatum in the general population remains unknown. This study examined the human seroprevalence of anti-Histoplasma antibody, and explored associations between seropositivity, and demographic and environmental variables, in Busia county, western Kenya.<br>Methodology<br>Biobanked serum samples and associated metadata, from a previous cross-sectional survey, were examined. Latex agglutination tests to detect the presence of anti-Histoplasma antibody were performed on serum samples from 670 survey respondents, representing 178 households within 102 sub-locations.<br>Potential epidemiologic risk factors for H. capsulatum exposure were explored using multi-level multivariable logistic regression analysis with household and sub-location included as random effects.<br>Principal findings<br>The apparent sample seroprevalence of anti-Histoplasma antibody was 15.5% (n=104/670, 95% Confidence Interval (CI) 12.9-18.5%). A multivariable logistic regression model identified increased odds of H. capsulatum seropositivity in respondents reporting rats within the household within the previous 12 months (OR=3.03 95% CI 1.06-8.67, p=0.04). Compared to respondents aged 25-34 years, the odds of seropositivity were higher in respondents aged 15-24 years (OR=2.66 95% CI 1.03-6.89, p=0.04) or ≥45 years (OR=2.64 95% CI 0.99-7.01, p=0.05).<br>Conclusions<br>The seroprevalence result provides a baseline for sample size approximations for future epidemiologic studies of the burden of H. capsulatum exposure in Busia county. The final model explored theoretically plausible risk factors for H. capsulatum exposure in the region. A number of factors may contribute to the complex epidemiological picture impacting H. capsulatum exposure status at the human-animal-environment interface in western Kenya. Focussed H. capsulatum research is warranted to determine the contextual significance of identified associations, and in representative sample populations.
**Additional Information:**	
**Question**	**Response**
**Government Employee**<br><br>Are you or any of the contributing authors an employee of the United States government?<br><br>Manuscripts authored by one or more US Government employees are not	No - No authors are employees of the U.S. government.

Powered by Editorial Manager® and ProduXion Manager® from Aries Systems Corporation

copyrighted, but are licensed under a CC0 Public Domain Dedication, which allows unlimited distribution and reuse of the article for any lawful purpose. This is a legal requirement for US Government employees.<br><br>This will be typeset if the manuscript is accepted for publication.	
**Financial Disclosure**<br><br>Enter a financial disclosure statement that describes the sources of funding for the work included in this submission and the role the funder(s) played. This includes grants and any commercial funding of the work or authors.<br><br>This statement will be typeset if the manuscript is accepted for publication.<br><br>Review the submission guidelines and the instructions link below for detailed requirements and guidance.	The original samples described were collected with the support of the Wellcome Trust (085308) and the Medical Research Council award. This work received support from the CGIAR Research Program on Agriculture for Nutrition and Health (A4NH), led by the International Food Policy Research Institute (IFPRI). Laboratory processing and analyses presented here were supported by funds from Scantlebury Wellcome Trust ISSF Fellowship award. The funders had no role in this study design, analysis, manuscript preparation or decision to publish. We also acknowledge the CGIAR Fund Donors (http://www.cgiar.org/about-us/our-funders).
**Competing Interests**<br><br>On behalf of all authors, disclose any competing interests that could be perceived to bias this work.<br><br>This statement will be typeset if the manuscript is accepted for publication.<br><br>Review the instructions link below and PLOS NTDs' competing interests policy to determine what information must be disclosed at submission.	There are no competing interests to declare.
**Data Availability**<br><br>Provide a **Data Availability Statement** in the box below. This statement should detail where the data used in this submission can be accessed. This	The original dataset, and the serology results, are available via an open access repository held by the University of Liverpool (http://dx.doi.org/10.17638/datacat.liverpool.ac.uk/352).

statement will be typeset if the manuscript is accepted for publication.<br><br>Before publication, authors are required to make all data underlying their findings fully available, without restriction. Review our PLOS Data Policy page for detailed information on this policy. Instructions for writing your Data Availability statement can be accessed via the Instructions link below.	

Powered by Editorial Manager® and ProduXion Manager® from Aries Systems Corporation

Manuscript

Click here to access/download;Manuscript;T Cornell et al_Kenya metadata analyis_Submission 1.pdf

Full title

Evidence of *Histoplasma capsulatum* seropositivity and exploration of risk factors for exposure in Busia county, western Kenya: Analysis of the PAZ dataset

Short title

Histoplasma capsulatum seroprevalence and risk factors in western Kenya

Author names and affiliations

Tessa Rose Cornell[1*], Lian Francesca Thomas[1,2], Elizabeth Anne Jessie Cook[2], Gina Pinchbeck[1], Judy Bettridge[1,2,#a,#b], Lauren Gordon[1,#c], Velma Kivali[2], Alice Kiyong'a[2], Eric Maurice Fèvre[1,2], Claire Elizabeth Scantlebury[1]

[1]Institute of Infection, Veterinary and Ecological Sciences (IVES), University of Liverpool, UK

[2]International Livestock Research Institute (ILRI), Nairobi, Kenya

[#a]Current address: Natural Resources Institute, University of Greenwich, UK

[#b]Current address: Bristol Veterinary School, University of Bristol, UK

[#c]Current address: Cellmark Forensic Services, UK

*Corresponding author

Email: Tessa.Cornell@liverpool.ac.uk (TC)

Abstract

Background

Despite recognition of histoplasmosis as a disease of national public health concern in Kenya, the burden of *Histoplasma capsulatum* in the general population remains unknown. This study examined the human seroprevalence of anti-*Histoplasma* antibody~~, ~~and explored associations between seropositivity~~,~~ and demographic and environmental variables, in Busia county, western Kenya.

Methodology

Biobanked serum samples and associated metadata, from a previous cross-sectional survey, were examined. Latex agglutination tests to detect the presence of anti-*Histoplasma* antibody were performed on serum samples from 670 survey respondents, representing 178 households within 102 sub-locations.

Potential epidemiologic risk factors for *H. capsulatum* exposure were explored using multi-level multivariable logistic regression analysis with household and sub-location included as random effects.

Principal findings

The apparent sample seroprevalence of anti-*Histoplasma* antibody was 15.5% (*n*=104/670, 95% Confidence Interval (CI) 12.9-18.5%). A multivariable logistic regression model identified increased odds of *H. capsulatum* seropositivity in respondents reporting rats within the household within the previous 12 months (OR=3.03 95% CI 1.06-8.67, *p*=0.04). Compared to respondents aged 25-34 years, the odds of seropositivity were higher in respondents aged 15-24 years (OR=2.66 95% CI 1.03-6.89, *p*=0.04) or ≥45 years (OR=2.64 95% CI 0.99-7.01, *p*=0.05).

Conclusions

The seroprevalence result provides a baseline for sample size approximations for future epidemiologic studies of the burden of *H. capsulatum* exposure in Busia county. The final model explored theoretically plausible risk factors for *H. capsulatum* exposure in the region. A number of factors may contribute to the complex epidemiological picture impacting *H. capsulatum* exposure status at the human-animal-environment interface in western Kenya. Focussed *H. capsulatum* research is warranted to determine the contextual significance of identified associations, and in representative sample populations.

Author Summary

Despite recognition of histoplasmosis as a priority disease of public health concern in Kenya~~,~~ and an important AIDS-defining illness, there remains a paucity of research on this neglected fungal disease. Clinical and laboratory capacity for the diagnosis and treatment of histoplasmosis across Kenya is limited or unknown, and existing diagnostic and therapeutic techniques can be cost-prohibitive. In addition, the fragmentary nature of histoplasmosis research groups worldwide~~,~~ and the under- or over-representation of specific sociodemographic groups and geographic regions in outbreak reports and hospital-based case series~~,~~ have been acknowledged.

This study provides a first look at *Histoplasma capsulatum* seroprevalence in rural western Kenya~~,~~ and explores risk factors for exposure at this human-animal-environment interface. More broadly, these outcomes will help ~~to~~ quantify the burden of *H. capsulatum* in household and community environments, which may direct further research efforts~~,~~ and inform policy-makers on the prioritisation for clinical services and public health efforts with regards to histoplasmosis.

Introduction

The burden of *Histoplasma capsulatum* is sparsely documented in sub-Saharan Africa, including in Kenya where histoplasmosis has been recognised as a priority disease of national public health concern [1,2]. Histoplasmin skin sensitivity surveys conducted in a limited number of countries in sub-Saharan Africa~~,~~ have recorded test positivity rates between 0.0 and 35.0%~~,~~ in populations with variable demographic and clinical characteristics [3–11]. These findings indicate that *H. capsulatum* is present within this geographic region. However, further research is warranted to explore the factors contributing to varying prevalence between different geographic areas and environments, the risk factors for exposure and infection, and the incidence and clinical outcomes of histoplasmosis.

The limited research in this area is confounded by multiple barriers to the identification and management of human histoplasmosis, which comprise: (i) case under-reporting; (ii) case mis-diagnosis; (iii) limited access to clinical facilities for case diagnosis or treatment; (iv) limited access to anti-fungal treatments; (v) cost-prohibitive diagnostic or treatment methods; and (vi) poor definition of transmission routes and risk factors for exposure [12–15]. These barriers present a significant challenge to histoplasmosis surveillance, treatment and infection control~~,~~ and thus limit our understanding of how *H. capsulatum* exposure or infection impacts the Kenyan population.

A number of risk factors for histoplasmosis are widely acknowledged, however evidence of contextual factors relevant to sub-Saharan Africa remains limited. Disseminated histoplasmosis has been identified as a major AIDS-defining disease presentation of HIV-infected patients [16]. In contrast to disease course in immunocompetent hosts, which is typically characterised as asymptomatic and self-limiting [17], patients with *H. capsulatum* and HIV co-infection have demonstrated significant morbidity and mortality rates in the absence of appropriate treatment [18,19].

Occupational and recreational activities speculated to increase risk of aerosolisation and inhalation of infective *H. capsulatum* microconidia~~,~~ have been described in histoplasmosis case and outbreak reports~~,~~ and hospital-based case series. Tunnel work [20], land excavation [21], bat habitat exposure during cave and tunnel visits [22,23], and exposure to bird faeces and roosts [24,25]~~,~~ have been reported as plausible risk factors for *H. capsulatum* exposure. *H. capsulatum* has been identified in soil and water samples [26–28], ~~and in~~ bats [29], and bat and bird faeces [30–34]~~,~~ by direct microscopy, mouse inoculation and culture technique, or molecular detection. In the rural Kenyan context, humans can live in close proximity with domestic and wild animals, and previously recognised reservoirs of *Histoplasma* could be present within household environments.

The current study utilised serum samples and metadata previously collected during a cross-sectional household survey in Busia county, western Kenya [35] to explore levels of exposure to *H. capsulatum*. The primary objectives of the study were as follows:

- Estimate the human seroprevalence of anti-*Histoplasma* antibody in Busia county, using a latex agglutination test (LAT);
- Explore associations between *H. capsulatum* seropositivity, and demographic and environmental variables in Busia county; and
- Identify limitations in current metadata with regards to identifying the burden of *H. capsulatum* exposure in the Kenyan context~~,~~ and thus highlight future research activities required to address gaps in evidence.

Methods

Original study and ethical approval

A cross-sectional household survey was conducted from 2010-12 in Busia county, western Kenya, for the People, Animals and their Zoonoses (PAZ) project, supported by the Wellcome Trust [36]. Project

outputs included epidemiologic data on the prevalence of neglected zoonotic diseases amongst 2113 survey respondents, from randomly selected households stratified by sub-location (*n*=143) [35,37]. For complete methodology of household selection, refer to Fèvre *et al.* (2017) [35].

Ethical approval for serum sample collection and storage for future processing, was granted by the Kenya Medical Research Institute (KEMRI; SSC 1701). Permission to re-analyse metadata and test bio-banked serum samples was provided by KEMRI, and supported by Scantlebury Wellcome Trust ISSF Fellowship.

Serum sample selection

Survey respondents were selected from a sub-set of the PAZ dataset, representing respondents with available data on bat observation (dataset recording bat observation: *n*=670/942, 71.1%; original dataset: *n*=670/2113, 31.7%). The data sub-set was collected between May 2011 and July 2012. Serum samples were selected to include survey respondents reporting variable HIV status at the time of sampling (positive: *n*=48/670, 7.2%), and absence or presence of bats around the home during the 12 months prior to survey delivery (bats observed: *n*=348/670, 51.9%) (Table 1). All respondents with a HIV positive status and all those reporting the presence of bats were selected, in addition to systematically selecting every eighth respondent across the dataset. The selected sub-set of survey respondents represented 178 households within 102 sub-locations.

A positive HIV status was recorded in 7.2% (*n*=48/670) of respondents, with the highest prevalence of HIV positive status among respondents aged 35-44 years (*n*=18/670, 23.7%), and females (Female: *n*=33/350, 9.4%; Male: *n*=15/320, 4.7%).

Table 1. Selection of study respondents (n=670/942, 71.1%) from the household survey (Fèvre et al., 2017), characterised by HIV status (Positive/ Negative), and bat observation around the home in previous 12 months (Yes/ No).

		Bats observed around home in previous 12 months		
		**No***	**Yes**	**Total**
**HIV result**	**Negative**	292	330	622
	**Positive**	30	18	48
**Total**		322	348	670

* No (*n*=321) or not answered (*n*=1)

Serological testing

One IMMY® Latex Agglutination *Histoplasma* test was performed as per manufacturer guidelines for each thawed, heat-treated serum sample. In accordance with IMMY® guidelines, a graduated scale of reaction strengths was used to assign test results from negative (-) to four plus (4+). Positive and negative controls had to demonstrate 2+ or greater, and less than 1+ reaction strengths, respectively [38]. Samples assigned a 2+ or greater reaction strength were considered to be presumptive evidence of active or recent *H. capsulatum* infection [38].

The LAT provides a measure of agglutinating anti-*Histoplasma* antibody, predominant during the early IgM antibody response. Antibody responses in individuals with acute histoplasmosis have been characterised by an initial peak in IgM mean concentration at 14 to 27 days, before returning to pre-clinical levels by one year [39]. Thus, a positive reaction may be indicative of exposure or infection up to six months prior.

Seroprevalence estimation

The apparent prevalence of *H. capsulatum* seropositivity in the sample population was determined based on the IMMY® Latex Agglutination *Histoplasma* test results. True seroprevalence was estimated using published sensitivity and specificity values for a histoplasmin sensitised LAT~~,~~ of 62% and 97%, respectively [40]. Epitools interface and Clopper-Pearson (exact) test were employed to determine 95% Confidence Intervals (CIs) (https://epitools.ausvet.com.au/trueprevalence [41]).

Statistical analysis

Household survey metadata~~,~~ and LAT results~~,~~ were stored in a protected Microsoft® Excel file. Metadata variables were selected for analysis if identified as either~~,~~ an established risk factor for *H. capsulatum* exposure in current literature, or a theoretically plausible risk factor for exposure based on current evidence of *H. capsulatum* life cycle and transmission dynamics (Tables S1-S2).

Descriptive statistics were used to analyse respondent- and household level-characteristics of the selected sub-set of respondents~~,~~ and to compare this sub-set with the original sampled population. The Mann-Whitney U test was applied to compare distributions of categorical variables between the original sample and the sub-set~~,~~ and to determine the statistical significance of differences.

Univariable associations between *H. capsulatum* seropositivity, and individual selected variables, were examined using Pearson Chi-squared test (χ^2). Odds Ratios (ORs) with 95% CIs, and associated *p*-values, were calculated.

Phi coefficient was employed to analyse suspected correlations between categorical variables. A coefficient value of >0.5, with an associated *p*-value <0.05, was interpreted as evidence of a correlation between variables. Identification of a correlation, and subsequent comparison of *p* values on univariable analysis, supported exclusion of variables from further analysis, where those with stronger *p*-values were retained for further analysis.

Variables with a χ^2-associated *p*-value <0.20 on univariable analysis were selected for testing in a multivariable logistic regression model with seropositivity as the binary outcome. The model was built using a manual backwards-stepwise approach. As the study is exploratory, and designed to generate hypotheses about potential risk factors for *H. capsulatum* exposure, a conservative cut-off value of *p*<0.10 was applied to include variables in the final model. Final versions of the model were assessed using the Hosmer Lemeshow test statistic, and Delta Betas were explored for variables within the final model to examine the effect of any influential data points. Random effects were included to explore the effect of clustering of respondents at both household, and sub-location levels. Regression coefficients, estimate *p*-values, and z-ratios were compared between single and multi-level models. Proportion of variance attributed to individual levels was calculated using the latent-variable approach described by Goldstein *et al.* (2002) [42].

Statistical analyses, and multi-level modelling, were performed using IBM® SPSS® Statistics 25, and MLwiN 3.05 software, respectively.

Results

Study population

Selected respondents (*n*=670/942, 71.1%) represented 178 households, with a median number of occupants of 7.0 per household (range: 1-30). The sample comprised respondents aged 5 to >85 years, and displayed a positively skewed age distribution (Fig. S1). Modal and median age categories were 5-14 years (*n*=272/670, 40.6%), and 15-24 years (*n*=133/670, 19.9%), respectively. The sample comprised 350 females (52.2%). The gender ratio per age category was approximately 90-125 females per 100 males, with the exception of age category 25-34 years which demonstrated a greater

gender gap of 213 females per 100 males. The majority of respondents were teachers or students (*n*=341/670, 50.9%)~~,~~ or within animal management or contact roles (*n*=244/670, 36.4%). A minority of respondents reported smoking behaviour (*n*=17/670, 2.5%) (Tables S3-S4).

Contact with dogs (*n*=582/670, 86.9%), cats (*n*=563/670, 84.0%), and poultry (*n*=597/670, 89.1%), and observation of rats (*n*=609/670, 90.9%)~~,~~ in and around the household environment~~,~~ were reported by the majority of respondents. Examination of indirect animal contact activities~~,~~ demonstrated that the majority of respondents were involved in manure preparation (*n*=436/670, 65.1%), in contrast to animal burial and skinning activities which were not frequently reported (Table S4). The most common manure preparation activities were described as~~,~~ preparation for fuel~~,~~ and use as a building material.

At a household level (*n*=178 households), spring water (wet: *n*=93/178, 52.2%; dry: *n*=92/178, 51.7%) and borehole sources (wet: *n*=73/178, 41.0%; dry: *n*=74/178, 41.6%) were most frequently reported in the previous wet and dry seasons. Houses within the study area tended to be constructed of: iron (*n*=128/178, 71.9%) or thatch (*n*=121/178, 68.0%) roofs; mud walls (*n*=160/178, 89.9%); and earth floors (*n*=156/178, 87.6%). The majority of households reported using firewood as the main cooking fuel (*n*=136/178, 76.4%) (Table S4).

No statistically significant differences were found in the demographic and behavioural variables between the original sample and the sub-set of respondents selected for this study, with the exception of observation of bats around the home which was significantly higher in the sub-set (p<0.001) due to the selection criteria applied for sub-setting (Table S5). We therefore consider the sub-set selected for this study to be appropriately representative of the underlying population, enabling population inferences to be made with regards to seroprevalence and risk factors being explored.

Seroprevalence results

A total of 104 serum samples were interpreted as positive on LAT (a reaction strength of 2+ or greater), of which the majority displayed a reaction strength of 2+ (*n*=68/104, 65.4%) (Table S6 and Fig. S2). This related to an apparent seroprevalence of 15.5% (*n*=104/670, 95% CI 12.9-18.5%). The estimated true seroprevalence in this sample, adjusting for published LAT sensitivity and specificity results [40], was calculated as 21.2% (95% CI 16.8-26.2%) [41].

43.3% of households (n=77/178) contained at least one occupant with a seropositive result. Of these households, the percentage of occupants demonstrating seropositivity ranged from 7.7% (n=1/13 occupants) to 100.0% (n=1/1 to 3/3 occupants) (Fig. 1).

Figure 1. Study area in Busia county, western Kenya, and household locations (*n*=178) within 102 sub-locations. Key: red = households with ≥1 occupant seropositive for *H. capsulatum*; blue = households with zero seropositive occupants. Maps generated using QGIS 3.12.2.

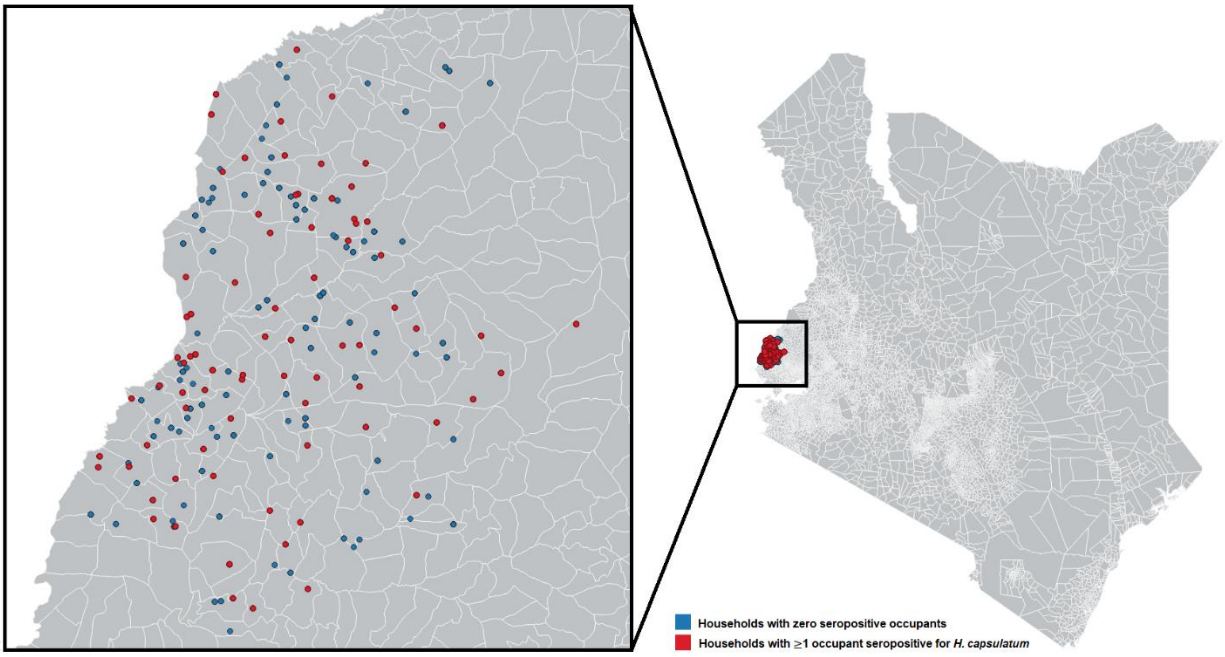

Univariable analysis

Univariable logistic regression analysis identified a statistically significant association (p<0.05) between LAT result and two variables; observation of rats around the home in the previous 12 months (OR=2.80 95% CI 0.99-7.89, p=0.05); and age category 15-24 years (OR=2.80 95% CI 1.10-7.15, p=0.03; reference category 25-34 years). Variables which met the multivariable model inclusion criteria at a higher p-value cut-off (p<0.1) were as follows; observation of bats in the previous 12 months (OR=1.37 95% CI 0.90-2.10, p=0.14), household constructed of mud walls (OR=2.26 95% CI 0.80-6.43, p=0.13), use of spring water in the previous dry season (OR=1.47 95% CI 0.95-2.28,

p=0.08), and age category ≥45 years (OR=2.41 95% CI 0.92-6.28, *p*=0.07; reference category 25-34 years) (Table S4).

For each water source variable, a statistically significant correlation (phi >0.5, *p*<0.05) was measured between reporting the use of the water source in the last wet season, and reporting use of the same water source in the last dry season. Thus, only water source variables for the last dry season, specifically tap, spring, well, river and borehole sources, were included in further analyses, and the seasonal element of the variable was excluded. Findings from univariable logistic regression analysis are available in Table S4.

Multivariable logistic regression analysis

The final multi-level multivariable model contains two statistically significant main effects; observation of rats around the home in previous 12 months (OR=3.03 95% CI 1.06-8.67, *p*=0.04), and age categories 15-24 (OR=2.66 95% CI 1.03-6.89, *p*=0.04; reference category 25-34 years) and ≥45 years (OR=2.64 95% CI 0.99-7.01, *p*=0.05), as variables associated with presence of anti-*Histoplasma* antibody (Table 2). The final model also included variables which met the inclusion criteria at a *p*-value cut-off of <0.1, as follows; bats observed around the home in the previous 12 months (OR=1.39 95% CI 0.89-2.16, *p*=0.14); and mud walls in the household (OR=2.67 95% CI 0.90-7.91, *p*=0.08) (Table 2).

Table 2. Multivariable logistic regression analysis examining variable associations with *H. capsulatum* seropositivity based on LAT results, amongst survey respondents (*n*=670/942, 71.1%) in Busia county, western Kenya. Odds Ratios (OR), 95% Confidence Intervals (CIs) and *p*-values, were calculated using MLwiN 3.05 software.

Variable	*H. capsulatum* seropositive (%), *n*=104	Odds Ratio (95% Confidence Intervals)	*p*-value
**Rats observed around home in previous 12 months**			
Reference category: No	4 (6.6)	1.00	
Yes	100 (16.4)	3.03 (1.06-8.67)	0.04*
**Bats observed around home in previous 12 months**			
Reference category: No	43 (13.4)	1.00	
Yes	61 (17.5)	1.39 (0.89-2.16)	0.14**
**Mud walls**			
Reference category: No	4 (7.8)	1.00	
Yes	100 (16.2)	2.67 (0.90-7.91)	0.08**

**Age category, years**			
5-14	38 (14.0)	1.74 (0.69-4.34)	0.24
15-24	27 (20.3)	2.66 (1.03-6.89)	0.04*
Reference category: 25-34	6 (8.3)	1.00	
35-44	12 (15.8)	2.13 (0.74-6.14)	0.16
≥45	21 (17.9)	2.64 (0.99-7.01)	0.05*

**p*-value <0.05 (statistically significant)

***p*-value <0.1

The model yielded a Hosmer-Lemeshow chi-squared value of 2.091 (*p*=0.978). On delta beta analysis, no data points were determined to be influential on the model outcome.

No evidence was demonstrated for clustering by household (variance=0.00, SE 0.00) on multi-level analysis. Clustering by sub-location was demonstrated (variance=0.16, SE 0.17) although this indicated that only 4.6% of variance in seropositivity is due to sub-location, using the latent-variable approach. Regression coefficients, z-ratios and *p*-values, of model variables in single-, two- and three-level models were comparable.

Discussion

The study describes the human seroprevalence of anti-*Histoplasma* antibody, and explores associations between seropositivity and potential risk factors for *H. capsulatum* exposure in a community and household setting, in Busia county, western Kenya.

The recent recognition of histoplasmosis as a priority disease in Kenya [1], and apparent seroprevalence of *H. capsulatum* exposure demonstrated by survey respondents (*n*=104/670, 15.5%), highlights the need for surveillance at national and regional levels. A previous histoplasmin skin test survey in Kenya reported a positivity rate of 8.5% (*n*=65/768) in adult males [9]. The age distribution of the study population was not reported, and participants were miners or prisoners from Lake Victoria (western Kenya), and within or west of the Rift Valley, respectively. Skin test positivity is lower than our apparent measured seroprevalence of 15.5% (*n*=104/670) which could be attributed to variable environmental conditions influencing survival of the saprophytic mycelial form of *H. capsulatum*, or variable exposure risk factors in the study populations under examination, including contact with animal reservoirs. In addition, the study described employs a histoplasmin skin sensitivity test as

opposed to the LAT described in this study, which measure IgE-mediated reactions versus IgM agglutinating antibody responses, respectively.

With the exception of case reports [43–48], and limited prevalence studies in select socio-demographic groups [9,49,50], there is a paucity of recent epidemiologic data examining the burden of *H. capsulatum* exposure in the general population, and in variable community and household settings, in Kenya, the surrounding region, and more widely across sub-Saharan Africa. In Nigeria, two cross-sectional studies examining histoplasmin skin sensitivity across variable regions, demonstrated positive tests in 4.4% (*n*=32/735) [8] and 10.5% (*n*=69/660) [6] of participants. The latter study was conducted in proximity to a bat cave, and a sub-sample of this study population identified as farmers, cave guides and traders in the vicinity of the cave (35.0%, *n*=14/40). Thus, the higher overall test positivity measured could be attributed to these study design factors.

Variables tested in the univariable and multivariable logistic regression models encompassed both established, and theoretically plausible, epidemiologic risk factors for *H. capsulatum* exposure. A significant association was identified between *H. capsulatum* seropositivity and the observation of rats within the household (OR=3.03 95% CI 1.06-8.67, *p*=0.04). In Kenya, *H. capsulatum* has been isolated from soil, including samples enriched with chicken and bat faeces [51–53]. Although evidence exists for the role of rats as environmental reservoirs, current literature is limited to North America, where *H. capsulatum* was identified in wild rats, and soil samples proximal to rat burrows [26,54,55]. Additional research is warranted in the community setting in western Kenya to explore any associations between *H. capsulatum* exposure and, the following variables: frequency and routes of human exposure to rats and their habitats, the location of rat burrows, isolation of *H. capsulatum* from rats and rat burrows, and the household and environmental factors maintaining rat populations.

The multivariable model presents associations ($p<0.1$) between, *H. capsulatum* seropositivity and both, the observation of bats around the home, and housing constructed with mud. Bat habitat exposure has been reported as a risk factor for *H. capsulatum* exposure [22,23], and *H. capsulatum* has been isolated from bats using molecular techniques [29]. This model suggests that bat or rat contact might increase risk of *H. capsulatum* exposure. The purposeful selection of respondents reporting observation of bats around the home should be considered, which might increase overall seropositivity compared to a randomly selected sample. The difference between distributions of

respondents reporting observation of bats in the study sample, and in the original sub-set of respondents, was statistically significant. Further investigation is warranted to examine the role of rats, bats and environmental reservoirs of *H. capsulatum* within this context. In addition, studies employing molecular methods, may support current literature on phylogenetic characterisation of *Histoplasma* isolates, and comparison to regional and global isolates from human, animal and environmental sources [56–58].

Exploration of potential associations between building materials, and the isolation of *H. capsulatum* in the household environment is warranted. One might hypothesise that mud walls may provide better substrate to maintain the saprophytic mycelial form of *H. capsulatum*, in comparison to brick or cement. Furthermore, different building construction methods might present variable *H. capsulatum* exposure risks, for example construction of mud walls with handheld tools might increase exposure risk from soil. The variables presented may also be proxy indicators of socioeconomic factors that increase risk of *H. capsulatum* exposure, and could be indicative of the sociodemographic differences between regions, and availability of building materials.

The multivariable logistic regression model demonstrates increased odds of seropositivity amongst age categories 15-24 (OR=2.66 95% CI 1.03-6.89, p=0.04) and ≥45 years (OR=2.64 95% CI 0.99-7.01, p=0.05), in comparison to respondents aged 25-34 years. Investigation of whether the outcome reflects variable immunocompetence between age categories, or age-related exposure to potential risk factors, is warranted. The sub-set of household survey respondents under examination demonstrated a positively skewed age distribution. At the time of data collection, in 2010, the age group 0-14 years represented 43.4% of the general Kenyan population [59]. This proportion is comparable to that of the sample population, of which 40.6% of selected respondents were 5-14 years (n=272/670). Although representative of the general population, the effect of a skewed population structure on the frequency distribution of other variables under investigation, including reported occupations and involvement in animal contact roles, should be considered. For example, these variables may not be sufficiently powered to explore risk factors in older age categories. In comparison to studies exploring demographic or clinical risk factors for *H. capsulatum* infection in susceptible patient cohorts, the current study highlights potential environmental risk factors amongst the general population which may be confounded to a lesser extent by age.

Further targeted research is warranted to explore the impact of potential confounders such as age, gender and occupation. Investigation of associations between dwelling maintenance activities, building materials including mud walls, and presence of wild or domestic animals in occupied dwellings, would provide further objective insight into the interactions of described household and environmental variables, and their impact on *H. capsulatum* exposure risk.

Although the described associations do not infer direct causality, nor encompass the unknown lifestyle and socioeconomic confounding factors, the variables presented contribute to the complex epidemiological picture influencing *H. capsulatum* exposure status at the human-animal-environment interface in western Kenya.

There was no evidence for significant clustering at household- nor sub-location levels, however further investigation is warranted to identify the potential socio-demographic and geoclimatic variations between defined areas that have not been explored in this analysis~~,~~ and to quantify their impact on the odds of seropositivity.

H. capsulatum was not a focus of the original PAZ study [35], thus questions posed by the survey were not designed to capture risk factors relating specifically to *H. capsulatum* exposure~~,~~ nor to capture temporal information which might be related to the timing of exposure. Factors that contribute to whether inhalation of *H. capsulatum* microconidia results in symptomatic disease~~,~~ include the quantity of airborne inoculum~~,~~ and the immunocompetence of the host. A robust T cell response and subsequent activation of macrophages can prevent progression of *H. capsulatum* infection [60], in contrast to the progressive nature of infection in immunocompromised individuals [61–63], however it should be noted that infection can become clinically apparent many years after first exposure.

With the exception of data on respondent smoking behaviour (yes: *n*=17/670, 2.5% [seropositive: *n*=4/17, 23.5%]) and HIV status (positive: *n*=48/670, 7.2% [seropositive: *n*=4, 8.3%]), clinical variables were excluded from analyses. The cross-sectional nature of data collection and absence of associated temporal data meant it was not possible to examine associations between reported clinical symptoms or disease and *H. capsulatum* seropositivity. Implementation of prospective, longitudinal research in community and household settings~~,~~ would enable more accurate inferences to be made about associations between *H. capsulatum* seropositivity~~,~~ and clinical signs or co-infections in the general population.

Hospital-based case series in Central and South America~~,~~ have examined morbidity and mortality in HIV-positive patients with confirmed disseminated histoplasmosis [64–66], and histoplasmosis is now widely recognised as a leading co-morbidity amongst AIDS patients [16]. An overall HIV prevalence of 7.7% of the general adult population was reported in Busia county in 2018 [67]. These individuals represent a potentially susceptible sub-set of the population to *H. capsulatum* co-infection. Clinical data with regards to individual immunocompetence of HIV-positive respondents at the time of survey delivery was not available, including access to and management of antiretroviral therapy. Among selected respondents, 48 (7.2%) demonstrated positive HIV status, however no statistically significant association was identified between HIV positive status and *H. capsulatum* seropositivity. An examination of the impact of HIV infection on immunodiffusion and complement fixation test results, revealed detection of anti-*Histoplasma* antibodies was significantly lower (p<0.05) in disseminated histoplasmosis cases with, as opposed to without, AIDS [68]. Thus, we speculate that measured seropositivity amongst HIV positive respondents in this study could be an underestimate due to the inability of these individuals to mount an immune response detectable by LAT. The effect of HIV co-infection on anti-*Histoplasma* antibody detection by LAT should be examined and quantified~~,~~ to improve our understanding of test performance and limitations~~,~~ and to increase the accuracy of seroprevalence estimates made on the basis of these test results. In addition, false positive results have been reported among patients with tuberculosis [69].

The possibility of cross-reactions with other systemic mycoses namely, *Aspergillus*, *Candida* and *Paracoccidioides* [70]~~,~~ should be acknowledged with the use of this LAT test. In the Kenyan context, *Aspergillus flavus* is documented as a major contaminant of maize crops, resulting in significant aflatoxin exposure [71]. Thus, the potential for cross-reactions with *Aspergillus*, specifically in a rural setting and in a maize-producing region, should be considered.

The LAT provides a measure of the presence of anti-*Histoplasma* antibody [38]. The IMMY® LA-*Histoplasma* test [38] references previously published overall sensitivity and specificity values [40]. However, test sensitivity ranged from 45.7 to 100%, for cases of chronic and acute primary pulmonary histoplasmosis, respectively [40]. Thus, the estimated true seroprevalence measured in the current study might vary significantly from 12.9% (95% CI 10.2-16.0%) to 29.3% (95% CI 23.2-36.2%) [41]. The samples were maintained temporarily at -20 degrees Celsius, prior to long term storage at -80

degrees Celsius. Freeze-thaw cycles were minimised and samples have not undergone any freeze-thaw cycles since 2016, thereby maintaining the integrity of samples for serological testing.

Conclusions

Results from the current study suggest that exposure to *H. capsulatum* occurs frequently within this population~~,~~ and promotes the need for further longitudinal research to investigate the incidence of *H. capsulatum* exposure and infection in Kenya. The seroprevalence reported here may provide a baseline for sample size approximations to support future epidemiologic studies of the burden of histoplasmosis.

Exploration of theoretically plausible risk factors has highlighted areas for further investigation. Future research might focus on further examination of the associations identified here, and consider how health, demographic, and socio-economic factors~~,~~ impact on *H. capsulatum* transmission at the human-animal-environment interface.

Acknowledgements

We thank the PAZ field and laboratory teams in Busia and Nairobi, Kenya, for their work in collecting the samples and metadata under analysis.

Funding

The original samples described were collected with the support of the Wellcome Trust (085308) and the Medical Research Council award. This work received support from the CGIAR Research Program on Agriculture for Nutrition and Health (A4NH), led by the International Food Policy Research Institute (IFPRI). Laboratory processing and analyses presented here were supported by funds from Scantlebury Wellcome Trust ISSF Fellowship award. The funders had no role in this study design, analysis, manuscript preparation or decision to publish. We also acknowledge the CGIAR Fund Donors (http://www.cgiar.org/about-us/our-funders).

Availability of data

The original dataset, and the serology results, are available via an open access repository held by the University of Liverpool (http://dx.doi.org/10.17638/datacat.liverpool.ac.uk/352).

References

1. Munyua P, Bitek A, Osoro E, Pieracci EG, Muema J, Mwatondo A, et al. Prioritization of Zoonotic Diseases in Kenya, 2015. PLoS One. 2016;11: e0161576. doi:10.1371/journal.pone.0161576

2. Kimanga D, Ogola S, Umuro M, Ng'ang'a A, Kimondo L, Murithi P, et al. Prevalence and incidence of HIV infection, trends, and risk factors among persons aged 15-64 years in Kenya: results from a nationally representative study. J Acquir Immune Defic Syndr. 2014;66 (Suppl. doi:10.1097/qai.0000000000000124

3. Ball JD, Evans PR. Histoplasmin sensitivity in Uganda. Br Med J. 1954; 848–849.

4. Bezjak V. Histoplasmin tests in Ugandan sawmill workers. Trop Geogr Med. 1971;23: 71–78.

5. Gugnani H, Egere J, Larsh H. Skin sensitivity to capsulatum and duboisii histoplasmins in Nigeria. J Trop Med Hyg. 1991; 24–6.

6. Muotoe-Okafor FA, Gugnani HC, Gugnani A. Skin and serum reactivity among humans to histoplasmin in the vicinity of a natural focus of Histoplasma capsulatum var. duboisii. Mycopathologia. KluwerAcademic Publishers; 1996.

7. Muotoe-Okafor FA, Gugnani HC, Gugnani A, Okafor G. Antibodies to antigens of Histoplasma, Blastomyces and Candida in HIV patients and carriers in Nigeria. Mycoses. 2000;43: 173–175. doi:https://doi.org/10.1046/j.1439-0507.2000.00568.x

8. Oladele RO, Toriello C, Ogunsola FT, Ayanlowo OO, Foden P, Fayemiwo AS, et al. Prior subclinical histoplasmosis revealed in Nigeria using histoplasmin skin testing. 2018 [cited 6 Aug 2021]. doi:10.1371/journal.pone.0196224

9. Stott H. Histoplasmin sensitivity and pulmonary calcification in Kenya. Br Med J. 1954; 22–25.

10. Lurie HI. Benign histoplasmosis: A preliminary report on the results of histoplasmin skin tests on residents of the Union of South Africa. South African Med J. 1949;23: 180–181.

11. Bezjak V, Farsey SJ. Prevalence of skin sensitivity to histoplasmin and coccidioidin in various Ugandan populations. Am J Trop Med Hyg. 1970;19: 664–669.

12. Oladele RO, Ayanlowo OO, Richardson MD, Denning DW. Histoplasmosis in Africa: An

emerging or a neglected disease? PLoS Negl Trop Dis. 2018;12. doi:10.1371/journal.pntd.0006046

13. Bongomin F, Kwizera R, Denning DW. Getting histoplasmosis on the map of international recommendations for patients with advanced hiv disease. J Fungi. 2019;5. doi:10.3390/jof5030080

14. Nacher M, Couppié P, Epelboin L, Djossou F, Demar M, Adenis A. Disseminated Histoplasmosis: Fighting a neglected killer of patients with advanced HIV disease in Latin America. PLOS Pathog. 2020;16: e1008449. doi:10.1371/journal.ppat.1008449

15. Adenis A, Nacher M, Hanf M, Vantilcke V, Boukhari R, Blachet D, et al. HIV-Associated Histoplasmosis Early Mortality and Incidence Trends: From Neglect to Priority. PLoS Negl Trop Dis. 2014;8. doi:10.1371/journal.pntd.0003100

16. Nacher M, Adenis A, Adriouch L, Dufour J, Papot E, Hanf M, et al. Short report: What is AIDS in the Amazon and the Guianas? Establishing the burden of disseminated histoplasmosis. Am J Trop Med Hyg. 2011;84: 239–240. doi:10.4269/ajtmh.2011.10-0251

17. Kauffman CA. Histoplasmosis: A clinical and laboratory update. Clinical Microbiology Reviews. 2007. pp. 115–132. doi:10.1128/CMR.00027-06

18. Adenis AA, Aznar C, Couppié P. Histoplasmosis in HIV-Infected Patients: A Review of New Developments and Remaining Gaps. doi:10.1007/s40475-014-0017-8

19. Lin J-S, Yang C-W, Wang D-W, Wu-Hsieh BA. Dendritic Cells Cross-Present Exogenous Fungal Antigens to Stimulate a Protective CD8 T Cell Response in Infection by Histoplasma capsulatum. J Immunol. 2005;174: 6282–6291. doi:10.4049/jimmunol.174.10.6282

20. Armstrong PA, Beard JD, Bonilla L, Arboleda N, Lindsley MD, Chae SR, et al. Outbreak of Severe Histoplasmosis among Tunnel Workers - Dominican Republic, 2015. Clin Infect Dis. 2018. doi:10.1093/cid/cix1067

21. Huhn GD, Austin C, Carr M, Heyer D, Boudreau P, Gilbert G, et al. Two outbreaks of occupationally acquired histoplasmosis: More than workers at risk. Environ Health Perspect. 2005;113: 585–589. doi:10.1289/ehp.7484

22. Gutierrez RE, Warnock DW, Bravo A V, Corella A, Rodriguez I, Lindsley MD, et al. Histoplasmosis associated with exploring a bat-inhabited cave in Costa Rica, 1998-1999. Am J Trop Med Hyg. 2004;70: 438–442. doi:10.4269/ajtmh.2004.70.438

23. Salomon J, Flament Saillour M, De Truchis P, Bougnoux ME, Dromer F, Dupont B, et al. An Outbreak of Acute Pulmonary Histoplasmosis in Members of a Trekking Trip in Martinique, French West Indies. J Travel Med. 2003;10.

24. Brodsky AL, Gregg MB, Loewenstein MS, Leo Kaufman Md, Mallison GF, Atlanta M. Outbreak of Histoplasmosis Associated with the 1970 Earth Day Activities. Am J Med. 1973;54: 333–342.

25. Waldman RJ, England AC, Tauxe R, Kline T, Weeks RJ, Ajello L, et al. A winter outbreak of acute histoplasmosis in Northern Michigan. Am J Epidemiol. 1983;117: 68–75. doi:10.1093/oxfordjournals.aje.a113517

26. Emmons CW. Isolation of Histoplasma capsulatum from soil. Public Health Rep. 1949;64: 892–896.

27. Gordon MA, Ajello L, Georg LK, Zeidberg LD. Microsporum gypseum and Histoplasma capsulatum Spores in Soil and Water. Science (80-). 1952;116.

28. Emmons CW. Isolation of Histoplasma capsulatum From Soil in Washington, D.C. Public Health Rep. 1961;76: 591–595.

29. Dos Santos B, Langoni H, da Silva RC, Menozzi BD, Bosco S de MG, Paiz LM, et al. Molecular detection of Histoplasma capsulatum in insectivorous and frugivorous bats in Southeastern Brazil. Med Mycol. 2018;56: 937–940. doi:10.1093/mmy/myx138

30. Zeidberg LD, Ajello L. Environmental factors influencing the occurrence of Histoplasma capsulatum and Microsporum gypseum in soil. J Bacteriol. 1954;68: 156–159.

31. Zeidberg LD, Ajello L, Dillon A, Runyon LC. Isolation of Histoplasma capsulatum from Soil. Am J Public Health. 1952;42: 930–935.

32. Gugnani HC, Muotoe-Okafor FA, Kaufman L. A natural focus of Histoplasma capsulatum var. duboisii is a bat cave. Mycopathologia. Kluwer Academic Publishers; 1994.

33. McMurray DN, Russell LH. Contribution of bats to the maintenance of Histoplasma capsulatum in a cave microfocus. Am J Trop Med Hyg. 1982;31: 527–531.

34. Disalvo AF, Bigler WJ, Ajello L, Johnson J, Palmer J. Bat and Soil Studies for Sources of Histoplasmosis in Florida. Public Heal Reports. 2010;85: 1063. doi:10.2307/4594044

35. Fèvre EM, de Glanville WA, Thomas LF, Cook EAJ, Kariuki S, Wamae CN. An integrated study of human and animal infectious disease in the Lake Victoria crescent small-holder crop-livestock production system, Kenya. BMC Infect Dis. 2017;17: 1–14. doi:10.1186/s12879-017-2559-6

36. ILRI. People, animals and their zoonoses | International Livestock Research Institute. 2021 [cited 9 Aug 2021]. Available: https://www.ilri.org/research/projects/people-animals-and-their-zoonoses

37. Thomas LF. Epidemiology of taenia solium Cysticercosis in western Kenya. University of Edinburgh. University of Edinburgh. 2014. Available: https://www.lib.uwo.ca/cgi-bin/ezpauthn.cgi?url=http://search.proquest.com/docview/1557570627?accountid=15115%0A https://ocul-uwo.primo.exlibrisgroup.com/openurl/01OCUL_UWO/01OCUL_UWO:UWO_DEFAULT??url_ver=Z39.88-2004&rft_val_fmt=info:ofi/fmt:kev:mtx:disser

38. IMMY. LA-Coccidioides, LA-Histoplasma,and LA-Sporothrix Antibody Systems. 2018. Available: https://www.immy.com/latex

39. Chandler JW, Smith TK, Newberry WM, Chin TDY, Kirkpatrickt CH. Immunology of the Mycoses. II. Characterization of the Immunoglobulin and Antibody Responses in Histoplasmosis. [cited 2 Aug 2022]. Available: https://academic.oup.com/jid/article/119/3/247/2189101

40. Hill GB, Campbell CC. Commercially available histoplasmin sensitized latex particles in an agglutination test for histoplasmosis. Mycopathologia. 1962;18: 169–176.

41. Epitools. Estimated true prevalence and predictive values from survey testing. 2021 [cited 19 Aug 2021]. Available: https://epitools.ausvet.com.au/trueprevalence

42. Goldstein H, Browne W, Rasbash J. Partitioning Variation in Multilevel Models. Underst Stat. 2002;1: 223–231.

43. Macharia A-M, Walong EO. Disseminated histoplasmosis in a paediatric patient at a referral hospital in Nairobi, Kenya. Pan Afr Med J. 2019;34: 139. doi:10.11604/pamj.2019.34.139.20685

44. Pamnani R. Disseminated histoplasmosis diagnosed on bone marrow aspirate cytology: report of four cases. East Afr Med J. 2009;86.

45. Shah M V, Ogada T, Nsanzumuhire H. Case report of Histoplasma capsulatum pulmonary histoplasmosis in a Kenyan African. East Afr Med J. 1978;55: 438–41.

46. McClatchie S, Kung'u A. Intestinal histoplasmosis in a Kenyan African. East Afr Med J. 1969;46: 166–9.

47. Davies P. A fatal case of histoplasmosis contracted in Kenya. East Afr Med J. 1957;34: 555–7.

48. Gellert A. A case of histoplasmosis successfully treated with Amphotericin B. East Afr Med J. 1960;37: 501–7.

49. Lofgren SM, Kirsch EJ, Maro VP, Morrissey AB, Msuya LJ, Kinabo GD, et al. Histoplasmosis among hospitalized febrile patients in northern Tanzania. Trans R Soc Trop Med Hyg. 2012;106: 504–507. doi:10.1016/j.trstmh.2012.05.009

50. Bahr NC, Lee D, Stauffer WM, Durkin M, Cetron MS, Wheat LJ, et al. Seroprevalence of Histoplasmosis in Somali, Burmese, and Hmong Refugees Residing in Thailand and Kenya. J Immigr Minor Heal. 2018;20: 334–338. doi:10.1007/s10903-017-0595-3

51. Kipyegon KE, Musyoki SK, Kariuki SM. Detection of Histoplasma Capsulatum in Domestic Chicken Droppings in Olenguruone, Nakuru County. Eur Sci Journal, ESJ. 2017;13: 119. doi:10.19044/esj.2017.v13n15p119

52. Nyaga PN, Kaminjolo JS. Histoplasmosis in Kenya: isolation of Histoplasma capsulatum from cave soil. Kenya Vet. 1978;2: 39–41.

53. Al-Doory Y, Kalter SS. The isolation of Histoplasma duboisii and keratinophilic fungi from soils of East Africa. Mycopathologia. 1969;31.

54. Emmons CW, Ashburn LL. Histoplasmosis in Wild Rats: Occurrence and Histopathology. Public Health Rep. 1948;63: 1416–1422.

55. Emmons CW, Morlan HB, Hill EL. Histoplasmosis in Rats and Skunks in Georgia. Public Health Rep. 1949;64: 1423–1431.

56. Teixeira M de M, Patané JSL, Taylor ML, Gómez BL, Theodoro RC, de Hoog S, et al. Worldwide Phylogenetic Distributions and Population Dynamics of the Genus Histoplasma. PLoS Negl Trop Dis. 2016;10. doi:10.1371/journal.pntd.0004732

57. Sepúlveda VE, Márquez R, Turissini DA, Goldman WE, Matute DR. Genome Sequences Reveal Cryptic Speciation in the Human Pathogen Histoplasma capsulatum . MBio. 2017;8. doi:10.1128/mbio.01339-17

58. Kasuga T, Taylor JW, White TJ. Phylogenetic relationships of varieties and geographical groups of the human pathogenic fungus Histoplasma capsulatum darling. J Clin Microbiol. 1999;37: 653–663.

59. World Bank. Kenya | Data. In: The World Bank Group [Internet]. 2021 [cited 27 Aug 2021]. Available: https://data.worldbank.org/country/kenya

60. Newman SL. Cell-mediated immunity to Histoplasma capsulatum. Semin Respir Infect. 2001;16: 102–8.

61. Evrard S, Caprasse P, Gavage P, Vasbien M, Radermacher J, Hayette MP, et al. Disseminated histoplasmosis: case report and review of the literature. Acta Clin Belgica Int J Clin Lab Med. 2018. doi:10.1080/17843286.2017.1376454

62. Tajań J, Espasa M, Sala M, Navarro M, Font B, González-Mart́in J, et al. Case report: Disseminated infection by Mycobacterium sherrisii and Histoplasma capsulatum in an African HIV-infected patient. Am J Trop Med Hyg. 2013;88: 914–917. doi:10.4269/ajtmh.12-0572

63. Loulergue P, Bastides F, Baudouin V, Chandenier J, Mariani-Kurkdjian P, Dupont B, et al. Literature Review and Case Histories of Histoplasma capsulatum var. duboisii Infections in HIV-infected Patients. Emerg Infect Dis. 2007;13: 1647–1652.

64. Boigues BCS, Paniago AMM, Lima GME, Nunes M de O, Uehara SN de O. Clinical outcomes

and risk factors for death from disseminated histoplasmosis in patients with AIDS who visited a high-complexity hospital in campo Grande, MS, Brazil. Rev Soc Bras Med Trop. 2018. doi:10.1590/0037-8682-0369-2017

65. Ramos IC, Soares YC, Damasceno LS, Libório MP, Farias LABG, Heukelbach J, et al. Predictive factors for disseminated histoplasmosis in AIDS patients with fever admitted to a reference hospital in Brazil. Rev Soc Bras Med Trop. 2018;51: 479–84. doi:10.1590/0037-8682-0425-2017

66. Gutierrez ME, Canton A, Sosa N, Puga E, Talavera L. Disseminated Histoplasmosis in Patients with AIDS in Panama: A Review of 104 Cases. Clin Infect Dis. 2005;40: 1199–1202. doi:10.1086/428842

67. National Aids Control Council. Ministry of Health Kenya HIV Estimates Report 2018. 2018. Available: www.nacc.or.ke

68. Tobón AM, Agudelo CA, Rosero DS, Ochoa JE, De Bedout C, Zuluaga A, et al. Disseminated Histoplasmosis: A comparative study between patients with Acquired Immunodeficiency Syndrome and non-Human Immunodeficiency Virus-infected individuals. Am J Trop Med Hyg. 2018;73: 576–582. doi:10.4269/ajtmh.2005.73.576

69. Disalvo AF, Corbett DS. Apparent False Positive Histoplasmin Latex Agglutination Tests in Patients with Tuberculosis. J Clin Microbiol. 1976.

70. Oliveira dos Santos P, Messias Rodrigues A, Ferreira Fernandes G, Helena Marques da Silva S, Burger E, Pires de Camargo Z. Immunodiagnosis of Paracoccidioidomycosis due to Paracoccidioides brasiliensis Using a Latex Test: Detection of Specific Antibody Anti-gp43 and Specific Antigen gp43. PLoS Negl Trop Dis. 2015;9. doi:10.1371/journal.pntd.0003516

71. Islam MS, Callicott KA, Mutegi C, Bandyopadhyay R, Cotty PJ. Aspergillus flavus resident in Kenya: High genetic diversity in an ancient population primarily shaped by clonal reproduction and mutation-driven evolution. Fungal Ecol. 2018;35: 20–33. doi:10.1016/J.FUNECO.2018.05.012

Supporting information

Table S1. Variables selected for data analysis, from original survey human and household survey reports. [1]Analysis of observations of bats and wild birds only.

Table S2. Original survey human and homestead report text, and re-coding of selected variables. NR=not recorded; ND=not determined; NA=not applicable.

Table S3. Re-categorised occupations for statistical analysis.

Table S4. Univariable logistic regression analysis results, examining associations between *H. capsulatum* seropositivity based on LAT results, and respondent- and household-level variables, amongst survey respondents (*n*=670/942) in Busia county, western Kenya. Odds Ratios (OR), 95% Confidence Intervals (CIs) and *p*-values, were calculated using IBM® SPSS® Statistics 25 software. * $p<0.05$; ** $p<0.1$.

Table S5. Baseline characteristics of original sample (*n*=942) and selected sub-set (*n*=670) of survey respondents, and comparison of variable distribution differences using the Mann-Whitney U test. * $p<0.05$.

Table S6. The frequency distribution of IMMY® Latex Agglutination-*Histoplasma* test results for study respondents (*n*=670/942), categorised by reaction strength, and result interpretation.

Figure S1. Histogram demonstrating the frequency distribution of age categories (years) for study respondents.

Figure S2. IMMY® Latex Agglutination-*Histoplasma* test demonstrating positive control (left), negative control (centre), and serum sample yielding positive result with a reaction strength of 2+ (right).

Click here to access/download;Figure;T Cornell et al_Kenya metadata analyis_Fig 1_v2.pdf

Figure 1. Study area in Busia county, western Kenya, and household locations (*n*=178) within 102 sub-locations. Key: red = households with ≥1 occupant seropositive for *H. capsulatum*; blue = households with zero seropositive occupants. Maps generated using QGIS 3.12.2.

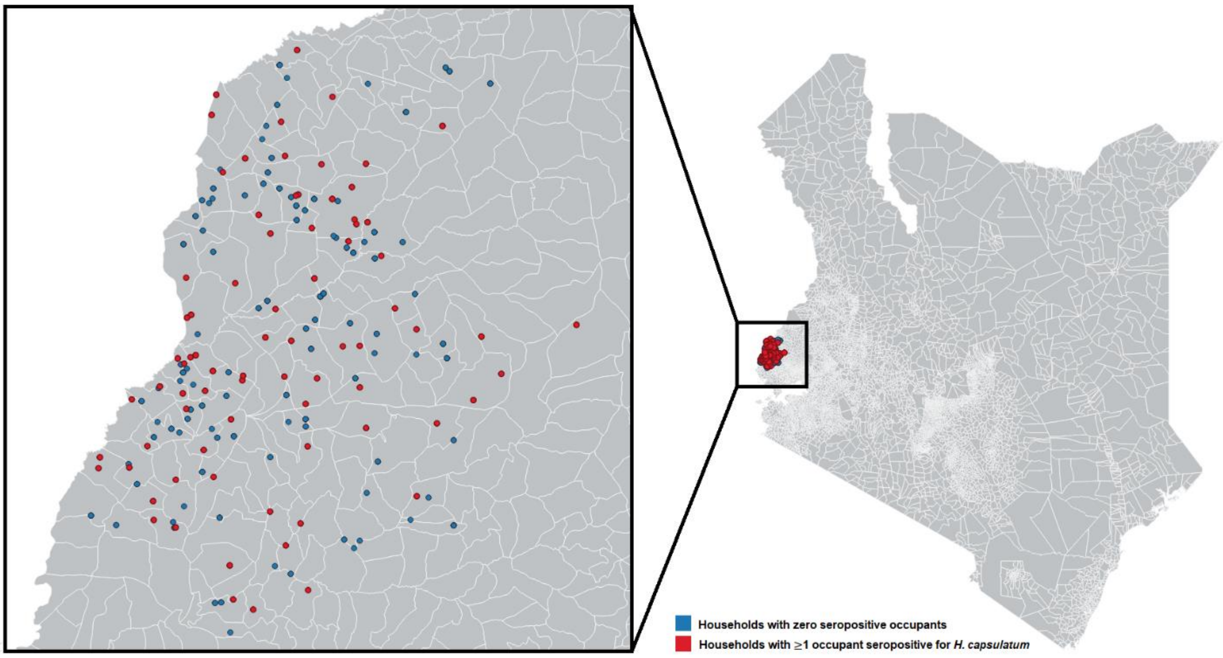

Table 1

Click here to access/download;Table;T Cornell et al_Kenya metadata analyis_Table 1.pdf

Table 1. Selection of study respondents (n=670/942, 71.1%) from the household survey (Fèvre et al., 2017), characterised by HIV status (Positive/ Negative), and bat observation around the home in previous 12 months (Yes/ No).

		Bats observed around home in previous 12 months		
		**No***	**Yes**	**Total**
**HIV result**	**Negative**	292	330	622
	**Positive**	30	18	48
**Total**		322	348	670

* No (*n*=321) or not answered (*n*=1)

Table 2 Click here to access/download;Table;T Cornell et al_Kenya metadata analyis_Table 2.pdf

Table 2. Multivariable logistic regression analysis examining variable associations with *H. capsulatum* seropositivity based on LAT results, amongst survey respondents (*n*=670/942, 71.1%) in Busia county, western Kenya. Odds Ratios (OR), 95% Confidence Intervals (CIs) and *p*-values, were calculated using MLwiN 3.05 software.

Variable	*H. capsulatum* seropositive (%), *n*=104	Odds Ratio (95% Confidence Intervals)	*p*-value
**Rats observed around home in previous 12 months**			
Reference category: No	4 (6.6)	1.00	
Yes	100 (16.4)	3.03 (1.06-8.67)	0.04*
**Bats observed around home in previous 12 months**			
Reference category: No	43 (13.4)	1.00	
Yes	61 (17.5)	1.39 (0.89-2.16)	0.14**
**Mud walls**			
Reference category: No	4 (7.8)	1.00	
Yes	100 (16.2)	2.67 (0.90-7.91)	0.08**
**Age category, years**			
5-14	38 (14.0)	1.74 (0.69-4.34)	0.24
15-24	27 (20.3)	2.66 (1.03-6.89)	0.04*
Reference category: 25-34	6 (8.3)	1.00	
35-44	12 (15.8)	2.13 (0.74-6.14)	0.16
≥45	21 (17.9)	2.64 (0.99-7.01)	0.05*

*p-value <0.05 (statistically significant)

**p-value <0.1

Figure S1

Click here to access/download
Supporting Information
T Cornell et al_Kenya metadata analyis_Fig S1.pdf

Figure S2

Click here to access/download
Supporting Information
T Cornell et al_Kenya metadata analyis_Fig S2.pdf

Table S1

Click here to access/download
Supporting Information
T Cornell et al_Kenya metadata analyis_Table S1.pdf

Table S2

Click here to access/download
Supporting Information
T Cornell et al_Kenya metadata analyis_Table S2.pdf

Table S3

Click here to access/download
Supporting Information
T Cornell et al_Kenya metadata analyis_Table S3.pdf

Table S4

Click here to access/download
Supporting Information
T Cornell et al_Kenya metadata analyis_Table S4.pdf

Table S5

Click here to access/download

Supporting Information

T Cornell et al_Kenya metadata analyis_Table S5.pdf

Table S6

Click here to access/download
Supporting Information
T Cornell et al_Kenya metadata analyis_Table S6.pdf
