# Supplementary material for: Evidence of Histoplasma capsulatum seropositivity and exploration of risk factors for exposure in Busia county, western Kenya: Analysis of the PAZ dataset
Source: PLoS Negl Trop Dis. 2023 May 12;17(5):e0011295. doi: 10.1371/journal.pntd.0011295 (PMC10180684; doi:10.1371/journal.pntd.0011295)
Supplement: Attachment — Submitted filename: T Cornell et al_Kenya metadata analysis_Responses_v2.pdf [file pntd.0011295.s008.pdf]

Dr Tessa Cornell

Institute of Infection, Veterinary and Ecological Sciences (IVES)

University of Liverpool, UK

Tessa.Cornell@liverpool.ac.uk

27 March 2023

Dear Dr Nosanchuk,

Cover letter: Responses to reviewer comments

Evidence of *Histoplasma capsulatum* seropositivity and exploration of risk factors for exposure in Busia county, western Kenya: Analysis of the PAZ dataset

Please find below the responses to reviewer comments and descriptions of changes made in the main manuscript. All line numbers, Tables or Figures referred to in the responses are as per the revised manuscript displaying tracked changes.

- *Line 96: Please include a word or two to define what kind of metadata this study investigated / collected.*
  - 'Metadata' changed to 'data' throughout the manuscript. The original data from participants are analysed so this is a more appropriate term.
  - Clarification on the type of data collection is made as follows (line 118-119): 'The current study utilised serum samples and data on demographic and animal exposure variables previously collected…'

- *Objectives are reasonably clearly stated. However, the text needs to make crystal clear with no ambiguity that the analysis in this paper explored associations of Histoplasma seropositivity and primarily non-clinical, demographic and environmental variables, so that no clinical conclusions should be drawn.*
  - This has been clarified as follows (line 123): 'Explore associations between *H. capsulatum* seropositivity, and non-clinical demographic and environmental variables in Busia county…'
  - Clarification was also added as follows (211-212): 'Data variables encompassed non-clinical demographic and environmental factors and…'

- *Line 108: It appears that Busia county of Western Kenya has been used as a surrogate for Rural Kenya. Please add a sentence or two in the Methods to indicate the reasons why Busia county was selected for running this study. Rural setting? Population? Zone of endemicity? Primary or pilot evaluation?*
  - Justification for selection of Busia county was made as follows (line 131-144): 'The study region was selected as it broadly represents the wider Lake Victoria Crescent ecosystem, namely that of a smallholder, mixed crop-livestock production system, with previously poorly understood burden of zoonotic infection [35].'

- *Line 118: Please add the absolute number of respondents here before going into the relative percentages.*
  - Edited as follows (line 153-154): 'A sub-set (*n*=942) of samples was selected from the PAZ dataset and represents respondents who received a question on bat observation…'

- *Line 121-122: When you say "serum samples were selected to include...", how do you mean? Your data is already a subset spanning 14 months. How were serum samples further selected?*

Were serum samples collected beyond the specified time frame brought into calculations simply based on their HIV status?

- The further selection criteria were applied to data collected during this time period.
- This is clarified as follows (line 155-156): 'Within this time period, the following criteria were applied to select the study sample of 670 survey respondents (*n*=670/942, 71.1%).'

- *Line 127 opening clause. This seems a duplication of the same information presented on lines 121-122.*
  - Moved to Results section (line 290-293).

- *Line 134: How does "not answered (n=1)" factor into the numbers in the table? Not answered what - HIV Status? Bats? Without this answer, how would that individual be incorporated into the data-generating cohort?*
  - Edited Table 1 to separate respondents answering No to bat question and respondent (*n*=1) who didn't respond to this question.
  - Wording of the selection criteria was edited as follows, to clarify that all respondents in the study dataset received the bat observation question and to encompass the participant that did not answer the question (line 153-154): 'A sub-set (*n*=942) of samples was selected from the PAZ dataset and represents respondents who received a question on bat observation.'
  - Edited Table 2 to clarify that respondent who didn't respond to questions on bat (or wild bird) observation was not included under 'No' category. This is reflected in the frequency (%) data columns.

- *'Lines 141-142: Please provide a better reference in support of the claim in this statement. Ref 38 is the IMMY booklet, which refers to a book chapter on Sporothrix schenckii. For the performance claims for the Histoplasma LA test, the IMMY booklet refers to a 1962(!) paper (your reference 40).*
  - Reference 38 is removed from this sentence. Justification for seropositivity with the LAT indicating active or recent exposure is described below with reference to further papers.

  My concerns are these:
  1) The IMMY LA test detects antibodies.
  - An edit is made as follows, to specify the LAT measures exposure, not infection (line 194-195): 'Samples assigned a 2+ or greater reaction strength were considered to be presumptive evidence of active or recent *H. capsulatum* <u>exposure</u>.'

  2) It takes generally 2-6 weeks to develop antibodies against Histoplasma; therefore, Ab tests may not detect acute infection even if the agglutination is primarily via IgMs.
  - This comment is addressed as follows, including an additional reference (line 200-203): '…Thus, a positive LAT reaction can indicate acute infection from 2 weeks post-exposure. Furthermore, IgM levels have demonstrated a significant decrease between acute and convalescent phases 5-6 and 10-12 weeks post-exposure, respectively [42]. Implications of these test limitations for estimating true seroprevalence are discussed further.'
  - A comment was added to the Discussion as follows (921-923): 'As the IgM antibody response to *Histoplasma* is mounted in 2-6 weeks, the LAT may present false negative results in individuals tested prior to 2 weeks post-exposure and after the IgM antibody response has diminished [39,41,42].'

  3) Immunosuppressed individuals may not develop a strong Ab response or may develop aberrant IgM responses, resulting in false negatives.
  - False negative results in immunosuppressed individuals is discussed as follows (line 895-901): 'Thus, we speculate that measured seropositivity amongst HIV positive respondents in this study could be an underestimate due to the inability of these individuals to mount an immune response detectable by LAT and subsequent false

negative results. The effect of HIV co-infection on anti-*Histoplasma* antibody detection by LAT should be examined and quantified to improve our understanding of test performance and limitations and to increase the accuracy of seroprevalence estimates made on the basis of these test results.'

ALSO, the IMMY LA test requires the serum to be titrated in a dilution series and reports a titer value that shows 2+ agglutination reaction. Please incorporate this information in your method statement.'

  - We only conducted one LAT per sample so the test was used only to report seropositivity in undiluted samples, not to report titer. This is clarified as follows (line 195-196): 'For the purpose of this study, no serum dilutions were performed as a measure of antibody titer.'
  - A comment was also added to the discussion as follows (915-917): 'Only one LAT was performed per serum sample for the purpose of this study. Serum dilutions could also be performed as a semi-quantitative measure of antibody titer.'

- *'Reference 39. Please note that this is a 1969 paper, which tested 5 male patients with histoplasmosis with LA test results available from 4 patients at 14-27 days after symptom onset. While this level of evidence was fine for 1969, a better reference is needed for 2023, especially when the same paper states that lymphocytes from patients with severe acute histoplasmosis may be impaired in their response to histoplasmin (the Ag that coats the latex particles in LA test).'*
  - As above, further references have been included. False negative test results in immunosuppressed individuals have also been discussed.

- *Statistical analysis. Please add 1 or more references for the statistical methods you have used. Line 172: Please provide a reference for your statistical model.*
  - Reference added for backward-stepwise approach to model building (line 249). This is a very standard approach to model building in logistic regression.

- *Line 163: Line 228. Please check in the title and all through if you meant "univariate" instead of "univariable" for the analysis. Regression models of all kinds (standard, logistic etc.) that involve a single outcome are referred to as "univariate" regardless of how many explanatory variables are included in the model.*
  Line 244. Please confirm that you mean to use "multivariable" instead of "multivariate"
  - Yes, these are univariable and multivariable models. We would ask the reviewer to check their comment. Multivariable model refers to analysis with one outcome (dependent) as we have in this study (and univariable with one explanatory variable and one outcome). Multivariate is used for the analysis with more than 1 outcome which is not the case here.

- *Line 164: You may have needed to construct 2x2 contingency tables with categorical variables in order to run the Chi-Squared test of independence / association and calculate Odds Ratios. If so, please indicate that in the Statistical methods.*
  - Edit made as follows (line 239-241): 'Univariable associations between *H. capsulatum* seropositivity and individual selected variables were examined by constructing 2xN contingency tables and using Pearson Chi-squared test of association (χ^2).'
  - Not all tables were 2x2 (some were 2xN) so this was stated.
- *Line 166: Phi coefficient is used to measure association between two categorical variables featuring a binary outcome. Please clarify if there was a step to convert all your categorical variable outcomes to a binary form.*
  - Re-coding of variables is described in 'Table S2 Original survey human and homestead report text, and re-coding of selected variables.' A comment is added, as follows (line 234): 'Re-coding of variables is described in Table S2'. Clarification is also added, as follows (line 242-243): 'Phi coefficient was employed to analyse suspected correlations between categorical variables <u>with a binary outcome</u>.'

ALSO, usually a Phi Coefficient of +0.7 to +1.0 is taken to indicate a strong positive association. Was there a reason to start at a weaker coefficient of 0.5 (perhaps to increase sensitivity of association tests)?

  - Coefficient edited to >0.7 (line 243 and 373). This revision didn't impact the results.

- *Line 168: Please confirm you meant "correlation" here (used for quantitative variables) as opposed to "association" (used for categorical variables).*
  - Edited 'correlation' to 'association' as appropriate.

- *The analytical approach is acceptable, and the results are reasonably well presented. However, there are two tables in the Supplementary Table list, which I think merit inclusion in the main manuscript. (I have more comments on this in the PDF annotations.)*
  Table S4. I am of the opinion that given the importance of this table, it should be presented in the main paper, not obscured as a supplementary table.
  Table S6 results should be summarized in text and presented within results.
  - Tables with LAT results and univariable logistic regression analyses moved to main manuscript.

  I would also like to see LAT images in which the + to +++ outcomes are clearly visible along with the controls for comparison. I think the composite LAT photo merits inclusion in the main manuscript.
  - Table 3 was edited to encompass descriptions of the LAT reaction strengths.
  - The LAT photo was moved to the main manuscript.
  - Reference images were added as Fig. S1.

- *Line 231. P=0.05 for rats (not surprising since the 95% CI includes 1) contradicts the statement on line 229 which asserts p<0.05 in the context of 2 variables, rats and young adult age.*
  - Edited description of rat variable as meeting the higher *p*-value cut-off (line 368-370): 'Variables which met the multivariable model inclusion criteria at a higher *p*-value cut-off (*p*<0.1) were as follows; observation of rats around the home in the previous 12 months (OR=2.80 90% CI 1.17-6.68, *p*=0.05)…'
  - Also, edit was made to Table 2 to clarify *p*-value for rat variable <0.1.

- *Line 234. Please confirm the use of 95% CI with a setting of P value of 0.1 (which usually indicates a CI of 90%)*
  - Edited 95% CIs to 90% CIs in uni- and multivariable analyses.

- *Table 2. Please clarify what the numbers under column H. capsulatum seropositive indicate, including ones in parentheses.*
  - Column headings have been revised in Tables 2 and 4. A column displaying seronegative participant frequencies (and percentages) per variable has been added to each table.

- *Table 2 data row 2: the p value is >0.1; why does it have ** which according to table footnote should be <0.1?*
  - Excluded bat variable from multivariable model to reflect *p*-value cut-off. Also, removed asterisks from bat variable p-value in Table 2 (as per revised manuscript) as appropriate.
  - A comment was made in the Discussion, as follows (line 794-795): 'The variable describing bat observation was not included in the final multivariable model. However, the…'
  - Bat and bat habitat exposure has been described in the literature as a risk factor for *Histoplasma* exposure, and respondents reporting bat observation were purposefully selected for this study sample, therefore the section describing this variable has been kept in the Discussion.

- *The authors have done a reasonably good job of presenting conclusions based on their research results. However, I am of the opinion that the authors need to revisit and clarify their statistical analyses (which underpin the conclusions), so that strong conclusions are not drawn based on weak associations.*
    - *P*-values were reviewed to ensure variables under review met the inclusion criteria and cut-offs stated in the methods. As above, bat variable in multivariable model was excluded as per *p*-value cut-off. As above, rat variable was maintained in final model but clarified that this variable met higher *p*-value cut-off in univariable analyses.
    - ORs and *p*-values were revised for variables in the multivariable model, following exclusion of the bat observation variable.
    - A comment was added to the Discussion, as follows (line 789-792): 'As stated, due to the exploratory nature of the study a conservative cut-off *p*-value was applied for inclusion of variables in the final model. Thus, strong conclusions should not be made based solely on these analyses and identified associations can contribute to generating hypotheses for future research.'
    - An edit was made to the Conclusions, as follows (line 933-934): 'Future research might focus on further examination of the significance of associations identified here…'

- Multi-level modelling was repeated as per revised model (excluding bat variable). Variance values due to household and sub-location were presented. Single- and multi-level models remained comparable.
    - Results were stated, as follows (line 552-554): 'Clustering by household (variance=0.02, SE 0.18) and by sub-location (variance=0.18, SE 0.17) were demonstrated. These outcomes indicated that only 0.6 and 5.2% of variance in seropositivity is due to household and sub-location respectively, using the latent-variable approach.'

- Hosmer-Lemeshow statistic was repeated for revised multivariable model.

- The Discussion section referring to potential associations with building materials has been moved, following removal of the bat variable from the final model (line 769-789).

- *Reference list. Please check individual references for completeness. Many are missing details from the bibliographic format.*
    - Reference format was reviewed.

- All comments regarding punctuation, spelling and terminology revisions (e.g. ethical to ethics) have been addressed.

- Edits to Table and Figure numbers in main manuscript and supplementary information have been made, following inclusion of extra Tables in main manuscript.

- Edits to formatting of Tables have been made in line with PLOS NTD guidelines.

- A link for the source of geodata used to produce Figure 2 has been stated (line 361-363).

Thank you again for your review of this manuscript.

Sincerely,

Tessa Cornell.
